# Low-Cost IoT-Based Computational System for Real-Time Biogas Monitoring in UASB Reactors Using NDIR Sensors and ESP32

**DOI:** 10.1021/acsomega.5c12291

**Published:** 2026-03-12

**Authors:** Flávio César Brito Nunes, Precival Victor Andrade Alves, Allan Bruno Dantas Gonçalves, Ricardo Cabral Lemos Filho, Joabesson Gonçalves Leandro da Silva, Vynicius Alves Calábria, Maria Gorethe de Sousa Lima Brito, Francisco José de Paula Filho

**Affiliations:** † Instituto Federal de Educação, Ciência e Tecnologia do Ceará, Juazeiro do Norte, CE 63047-040, Brazil; ‡ 423875 Universidade Federal do Cariri, Juazeiro do Norte, CE 63048-080, Brazil

## Abstract

UASB reactors are widely employed in wastewater treatment due to their operational simplicity and the potential for energy recovery from biogas, although continuous, low-cost monitoring of CH_4_ and flow rate remains challenging. This work presents the development and validation of an IoT system for remote, real-time monitoring, integrating NDIR sensors for CH_4_/CO_2_, a temperature sensor, a pressure sensor, a thermal mass flow meter, and an ESP32 platform with web/mobile interfaces. Deployment was carried out in a bench-scale UASB reactor treating an industrial slaughterhouse effluent. Over 30 days of continuous operation, stable data transmission was recorded with an average latency of ∼1.77 s; measurements covered 42.84–76.16 NL·d^–1^ (flow) and 53.31–88.0% (CH_4_), with temperature within a narrow mesophilic range (22.25–27.80 °C) and near-zero sensor drift. Estimates based on removed chemical oxygen demand (COD), normalized to STP, yielded 45.18–74.72 NL·d^–1^ (flow). Temporal agreement with the measured series was observed (MAE = 6.58 NL·d^–1^ and 4.69 percentage points; MAPE = 9.88% and 6.69% for flow and composition, respectively). This modular, fault-tolerant architecture demonstrates feasibility for supporting operational control and assessing the methane energy potential in decentralized applications.

## Graphical abstract

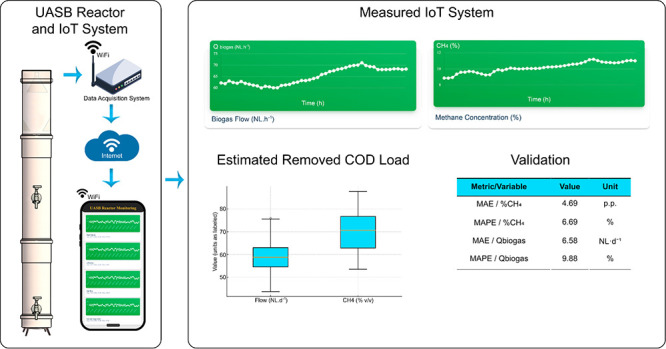

## 1 Introduction

Upflow anaerobic sludge blanket (UASB) reactors are a well-established technology for wastewater treatment, combining operational simplicity, high organic matter removal, and biogas generation with energy recovery potential.^1–3^ In decentralized contexts, however, continuous, low-cost monitoring of biogas production (flow rate) and composition (CH_4_/CO_2_) remains limited by the cost, infrastructure needs, and robustness of commercial solutions—especially for small units or facilities located in areas with intermittent connectivity.^4,5^ These limitations hinder the early detection of operational deviations, quantification of energy potential, and real-time decision-making.

The convergence of the Internet of Things (IoT), low-cost microcontrollers, and NDIR sensors has enabled accessible field-validated instrumentation. In particular, platforms such as the ESP32 integrate acquisition, processing, and wireless telemetry at costs substantially lower than proprietary analyzers while maintaining appropriate sampling cadence and fault tolerance.^6–8^ When combined with thermal flow meters and routines for temperature compensation and normalization to STP, such architectures provide operational estimates of biogas production and methane content with controlled error and good temporal adherence to process variations, meeting control and energy assessment requirements in wastewater treatment plants (WWTPs).^9–12^

This article addresses these gaps and presents a low-cost IoT system for remote, real-time monitoring of the biogas composition (CH_4_/CO_2_) and production (flow rate) in UASB reactors. The solution integrates NDIR sensors and flow measurement into an ESP32 node, with resilient firmware (watchdog, local buffering, and OTA), open-protocol communications, and a web/mobile backend for visualization and alerts.^7,8^ The system was validated in-plant, demonstrating communication stability, representative operating ranges, and temporal agreement between measured series and estimates derived from removed COD, with error metrics reported transparently.^9,10^

The main contributions are (i) a modular, scalable, and fault-tolerant architecture suitable for decentralized WWTPs, with stable telemetry and latency compatible with operational monitoring;^7^ (ii) operational validation with statistical analysis, covering CH_4_ and flow ranges typical of UASB reactors and demonstrating temporal adherence to process variations;^10^ (iii) integration with industrial sensors and protocols (NDIR via serial interface; flow via RS-485/Modbus), supported by embedded hardware implementation guidelines;^8^ and (iv) reproducible procedures for normalization to STP, calibration, and evaluation, facilitating comparability and replicability.^9,10^

By simultaneously addressing cost, connectivity, robustness, and field validation, the proposed approach brings low-cost IoT solutions closer to the level required to support operational control and energy valorization of biogas in real-world sanitation scenarios.^4,5^

## 2 Materials and Methods

### 2.1 General System Description

The monitoring system was developed using a low-cost embedded architecture composed of two ESP32 microcontrollers, NDIR gas sensors (for CH_4_ and CO_2_), environmental sensors (temperature and pressure), and a thermal mass flow meter. It is worth noting that differential pressure was monitored for operational diagnostics, but it was not used for data normalization or calculations in the analyses reported here, because the flow meter output was already provided as dry gas normalized to STP. The collected data are transmitted via Wi-Fi to a remote server and can be accessed in real time through a mobile application and a web interface. Figure 1 presents the flowchart of the system’s overall architecture.

**Figure 1:**
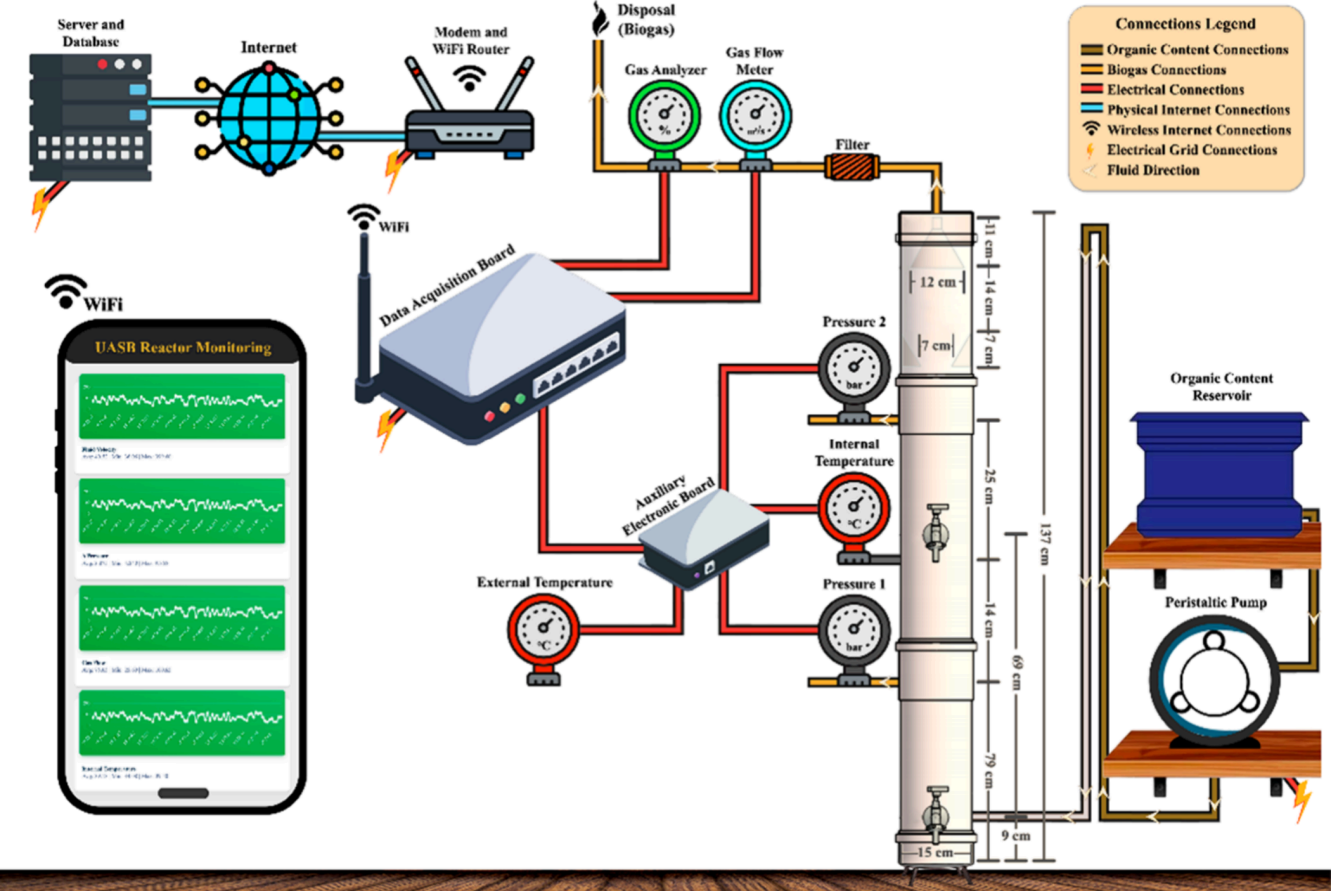
Flowchart of the overall architecture of the computational monitoring system.

### 2.2 Experimental Setup

The system was tested in a bench-scale UASB reactor with a working volume of 24 L, continuously fed with an industrial slaughterhouse effluent pretreated by screening, and a grit chamber. The flow rate was controlled by a peristaltic pump at 1.0 mL·s^–1^, and the operating temperature ranged from 23.3 to 26.0 °C.

### 2.3 Hardware and Sensors Used

The sensors and main devices employed are listed in Table 1, including the operating range and output type.

**Table 1:** Sensors Used in the Monitoring System

sensor	parameter	measurement range	output type
Gascard NG	CH_4_	0–100% v/v	RS-232/4–20 mA
Gascard NG	CO_2_	0–50% v/v	RS-232/4–20 mA
thermal mass flow meter	biogas mass flow rate	0–500 L·h^–1^	RS-485/4–20 mA
DS18B20	internal temperature	–55 to 125 °C	digital (single-wire proprietary protocol)
MPX5100DP	differential pressure	0–100 kPa	analog (0–5 V)
DHT22	ambient temperature	–40 to 80 °C	digital (single-wire proprietary protocol)

### 2.4 Data Acquisition and Operational Validation

The data acquisition system was implemented by using two ESP32 microcontrollers. The first was responsible for acquiring data from the thermal mass flow meter and the NDIR sensor and transmitting them via the I^2^C protocol to a second ESP32, which, in turn, acquired the remaining data from the environmental sensors and then sent the complete data set to the cloud server through a Wi-Fi connection. Readings were programmed to occur automatically every 30 min.

The temperature sensors were periodically compared with a reference instrument; when necessary, small offset adjustments were applied via the firmware.

The NDIR sensors (CH_4_/CO_2_) and the thermal mass flow meter were operated with factory calibration, and their validation was conducted at the operational level through two complementary approaches: (i) verification of the integrity and stability of data acquisition and transmission and (ii) comparison of the measured time series with theoretical estimates of biogas production derived from the removed organic load. The acquisition architecture employed dedicated serial communication for each instrument, with the flow meter integrated via Modbus RTU over RS-485, enabling the reading of instantaneous and accumulated flow registers, and the NDIR analyzer integrated via RS-232 serial stream. This configuration ensured continuous data acquisition and allowed assessment of the temporal and internal consistency of the signals.

For validation against theoretical estimates, methane production was estimated from the removed COD load, adopting a stoichiometric yield under standard conditions of 0.35 L CH_4_ per g of COD removed. The removed COD was obtained from BOD_5_ and influent flow rate, with BOD-to-COD conversion based on the factor defined in the study. From the estimated CH_4_ production, total biogas production was calculated using a methane volumetric fraction (*K*) adopted from the literature, thus maintaining independence between (i) the estimation of CH_4_ production from organic load removal and (ii) the assumed biogas composition.

These procedures supported the operational validation of the system based on coherence among measurement channels, stability of the data flow, and compatibility between measured values and theoretical estimates derived from organic load removal.

### 2.5 Mobile Application and Web Interface

An Android application was developed using the Flutter framework, featuring real-time readings, historical graphs, operational alarms, and data export in.csv format. Communication latency between data transmission by the ESP32 and server acknowledgment was monitored throughout the reliability and robustness tests (Section 2.6).

Additionally, a responsive web interface was developed (Figure 2), enabling data visualization in modern web browsers with a consolidated dashboard for real-time monitoring.

**Figure 2:**
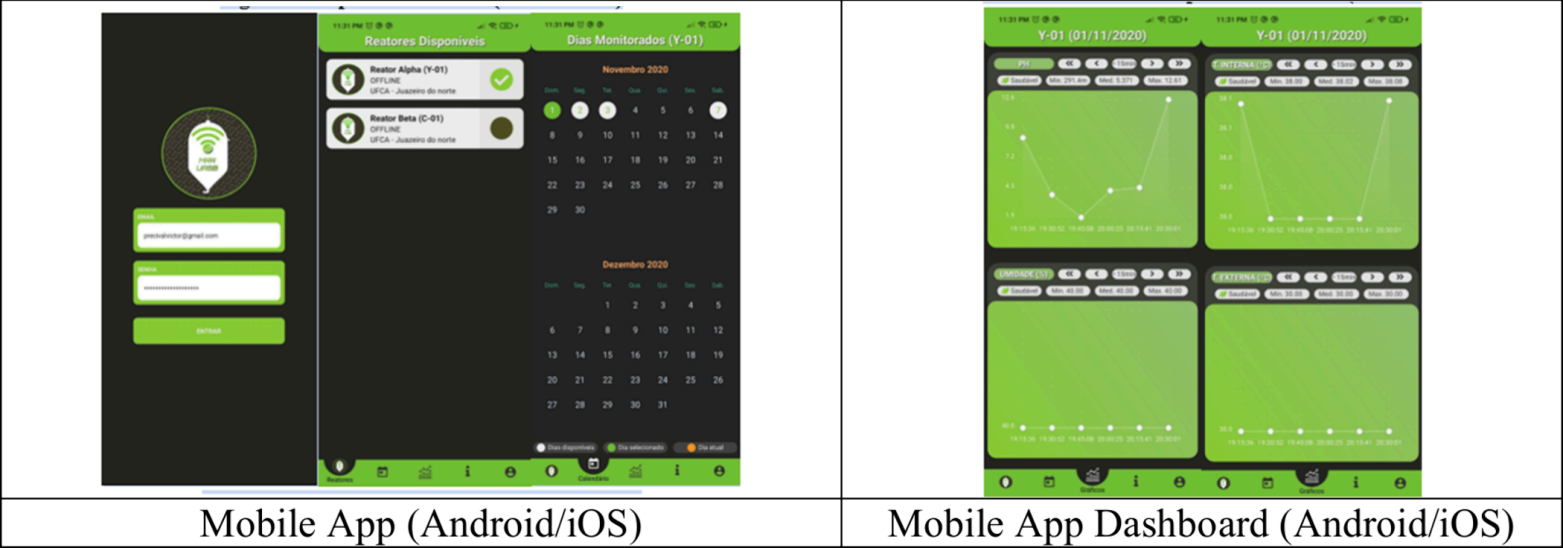
Monitoring interface.

### 2.6 Reliability and Robustness Tests

To validate communication and data storage, the system was subjected to different operating conditions. Long-duration trials (30 days) were conducted to verify the consistency of transmitted information and the successful sampling rate. A continuous seven-day experimental test was also carried out, with the sampling rate set to every 30 min (a total of 336 transmissions). In addition, Wi-Fi disconnections and router shutdowns were simulated, as well as power supply interruptions, in order to assess fault tolerance. Communication latency between data dispatch by ESP32 and server acknowledgment was monitored throughout the tests.

### 2.7 Estimation of Biogas Production and Methane Percentage

Methane production was estimated by applying the stoichiometric yield *k* = 0.35 L CH_4_·g COD^–1^(0 °C, 1 atm) to the removed COD load, with flow rates normalized to STP.^9,10^ The estimate of removed COD was derived from BOD_5_ (20 °C) and the influent flow rate, converting BOD to COD using a calibrated ratio *f* = 0.65 (±0.05) for the slaughterhouse effluent with high fat content, in order to prioritize the biodegradable fraction relevant to methanogenesis and reduce biases associated with using total COD in lipid-rich matrices; this choice showed better adherence to the measured biogas data.^2,13,14^

Total biogas was determined as the quotient between the estimated methane and the volumetric fraction of CH_4_ (*K*) adopted from the literature for high-fat effluents (45–75%).^4^ To avoid validation bias, *K* from the literature (or from an independent submodel) was used, keeping the production (CH_4_) and composition (%CH_4_) stages independent.^2,4^ Thus, the %CH_4_ estimated in this study derives exclusively from the compositional assumption represented by *K* rather than from an inference based on COD/BOD removal. From a physicochemical standpoint, the stoichiometry of the removed load allows estimation of the volume of CH_4_ generated; however, the volumetric fraction of CH_4_ in biogas depends on multiple factors, such as CO_2_ partitioning and solubility in the liquid phase, alkalinity and carbonate/bicarbonate equilibrium, stripping, water vapor presence, and hydrodynamic conditions, and therefore cannot be uniquely determined solely from the removed organic load. Accordingly, *K* was treated as an independent compositional parameter (dimensionless, 0 ≤ *K* ≤ 1), adopted within the typical range reported for this type of effluent, and used to (i) convert the estimated methane production into total biogas flow rate (eq 4) and (ii) directly express the estimated methane content on a volumetric basis (eq 5).^2,14^

In this context, the conversion from BOD_5_ to COD was performed according to eq 1:

$${\mathrm{COD}}_{\mathrm{in}/\mathrm{out}}\left[\mathrm{mg}\cdot L^{‐1}\right]=\frac{{\mathrm{BOD}}_{\mathrm{in}/\mathrm{out}}\left[\mathrm{mg}\cdot L^{‐1}\right]}{f}$$ 1

where COD_in/out_ is the influent/effluent COD, BOD_5 in/out_ is the influent/effluent BOD_5_, and *f* is the BOD-to-COD conversion factor.

The removed COD load (*L*_COD, rem_) was calculated using eq 2:

$$L_{\mathrm{COD},\mathrm{rem}}\left[g\cdot d^{-1}\right]=\left({\mathrm{COD}}_{\mathrm{in}}-{\mathrm{COD}}_{\mathrm{out}}\right)\left[\mathrm{mg}\cdot L^{-1}\right]\cdot Q_{l}\left[m^{3}\cdot d^{-1}\right]\cdot 10^{3}\left[g\cdot m^{-3}\right]$$ 2

where $Q_{l}$ is the influent flow rate and 10^3^ converts mg·L^–1^ to g·m^–3^.

The daily methane production (*Q*_CH_4__) was estimated from the removed COD load and the stoichiometric yield, as given by eq 3:

$$Q_{{\mathrm{CH}}_{4}}[\mathrm{NL}\cdot d^{-1}]=k[\mathrm{NL}\;{\mathrm{CH}}_{4}\;g\;{\mathrm{COD}}^{-1}]\cdot L_{\mathrm{COD},\mathrm{rem}}[g\;d^{-1}]$$ 3

where *k* is the stoichiometric yield.

Total biogas was obtained by dividing methane production by the methane volumetric fraction *K*, according to eq 4:

$$Q_{\mathrm{biogas}}[\mathrm{NL}\cdot d^{-1}]=\frac{Q_{{\mathrm{CH}}_{4}}}{K}$$ 4

where *Q*_biogas_ is the total daily biogas flow rate and *K* is the volumetric fraction of CH_4_ (typically 0.45–0.75).

$$K=y_{\mathrm{CH}4\;\mathrm{biogas}}$$ 5

where *K* is the methane volumetric (or molar) fraction and *y*_CH4_ is the methane fraction in biogas.

To avoid any ambiguity, *K* is defined as the volumetric (or molar) fraction of methane in the biogas (eq 5). Because biogas is treated here as an ideal gas mixture, the volumetric (or molar) fractions of its main constituents satisfy the closure condition in eq 6 (where *y*_tr_ represents trace gases). Consequently, the methane content expressed as % v/v follows directly from *K*, as shown in eq 7. Thus, in the present study, the estimated %CH_4_ corresponds to the percentage expression of the compositional assumption *K* adopted from the literature rather than to a value obtained by regression fitting or derived from the stoichiometry of the removed organic load. For the composition error metrics, a representative fixed value of *K* (*K* = 0.60; central within the literature range 0.45–0.75 for this type of effluent) was adopted to generate a reference %CH_4_^est^ time series (eq 7); MAE and MAPE were then computed by comparing %CH_4_ measured versus %CH_4_^est^ across all paired timestamps, avoiding circularity (i.e., without tuning *K* based on the measured %CH_4_). This choice preserves the independence between (i) the calculation of CH_4_ production from the removed load (eq 3) and (ii) the biogas composition assumption used to convert CH_4_ into total biogas (eq 4) and to express the estimated methane content (eq 7).

$$y_{\mathrm{CH}4}+y_{\mathrm{CO}2}+y_{\mathrm{tr}}=1$$ 6

where *y*_CH4_ is the methane fraction, *y*_CO2_ is the carbon dioxide fraction, and *y*_tr_ is the trace-gas fraction.

$$\%{\mathrm{CH}}_{4\;(v/v)}=100K$$ 7

where %CH_4_ is the methane content in percentage points and *K* is the methane volumetric fraction (0–1).

When necessary, gas volumes were normalized to STP considering pressure, temperature, and relative humidity, as given by eq 8:

$$V_{N}=V_{m}\frac{P}{P_{0}}\frac{T_{0}}{T}\left(1-\phi \frac{P_{\mathrm{ws}}(T)}{P}\right)$$ 8

where *V*_N_ is the normalized volume (STP); *V*_m_ is the measured volume; *P*, *T* are the pressure/temperature at measurement, respectively; *P*_0_, *T*_0_ are 1 atm and 273.15 K, respectively; ϕ is the relative humidity (0–1); and *P*_ws_(*T*) is the water vapor pressure at temperature *T*. If the system already reports dry gas normalized to STP, eq 8 is not applied.

### 2.8 Statistical Analysis

To describe data dispersion, an unpaired analysis was performed for each variable using all valid measurements within the observed ranges (flow rate: NL·d^–1^; CH_4_: v/v). The boxplots followed the Tukey convention (median; IQR = Q1–Q3; whiskers = 1.5 × IQR; outliers beyond the whiskers). We report *n*, median, IQR, mean, standard deviation (SD), and coefficient of variation (CV%).

To evaluate the performance of the stoichiometric–empirical model for biogas production and composition, the main metrics used were the mean absolute error (MAE) and the mean absolute percentage error (MAPE), according to eqs 9 and 10.

$$\mathrm{MAE}=\frac{1}{n}\overset{n}{\underset{i=1}{\sum }}\left|{ŷ}_{i}-y_{i}\right|$$ 9

$$\mathrm{MAPE}=\frac{100}{n}\overset{n}{\underset{i=1}{\sum }}\left|\frac{{ŷ}_{i}-y_{i}}{y_{i}}\right|$$ 10

For the biogas flow rate, MAE (NL·d^–1^) was prioritized in order to preserve the physical unit and avoid distortions at very low observed values, while MAPE was used only as a complementary metric. For the CH_4_ fraction, MAE in percentage points (p.p.) was emphasized, with MAPE maintained as an additional summary indicator.

Temperature results were subjected to descriptive statistics (*n*, mean, SD, CV%, median, and IQR). Stability or drift was estimated by regression analysis, expressed as slope (°C·day^–1^) with a 95% confidence interval and *p*-value, and residual autocorrelation was assessed using the Durbin–Watson statistic and the autocorrelation function (ACF). For method agreement based on ex situ measurements, strict temporal pairing was performed (±5–10 min window), followed by calculation of mean bias (±95% CI) and limits of agreement according to Bland–Altman.^16^

Metrological acceptance considered the DS18B20 specification (nominal accuracy ±0.5 °C between −10 and +85 °C; configurable resolution up to 0.0625 °C, 12 bits), while operational interpretation was anchored in the typical mesophilic range of anaerobic reactors and their expected effects on microbial metabolism.^2,9^

### 2.9 Hardware Development: Pressure and Temperature Data Acquisition Board

Using the MPX5100DP pressure sensors, the DHT22 temperature sensor, and the DS18B20 sensor for measuring the reactor’s internal temperature, an electronic board was built—based on an ESP32 microcontroller—for acquiring the reactor’s internal differential pressure as well as its internal temperature.

After preliminary laboratory tests, it was found that a single ESP32 microcontroller was insufficient to simultaneously read all sensors and continuously transmit data to the cloud storage platform via Wi-Fi. Given this limitation, a second ESP32 microcontroller was incorporated, dedicated exclusively to reading data from the thermal mass flow meter and the infrared gas characterization sensor board (Gascard NG). These data were then transferred to the first microcontroller via the I^2^C serial communication protocol, as illustrated in Figure 3a, b.

**Figure 3:**
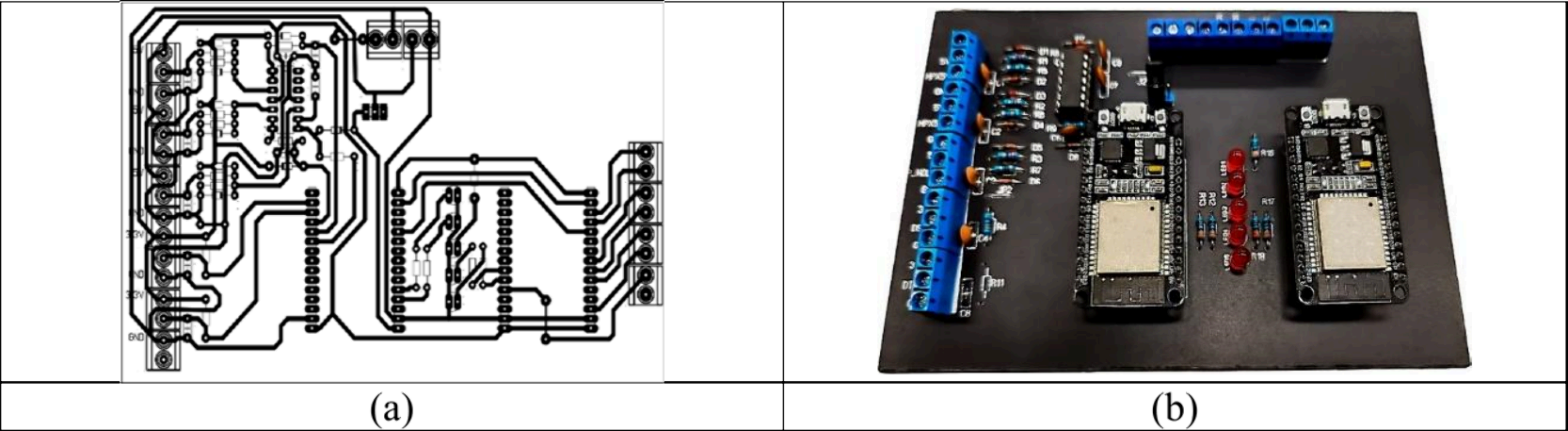
(a) Data acquisition board routing; (b) front view of the data acquisition board.

Given that the physicochemical characteristics of the UASB reactor require a significant interval to exhibit meaningful variations, a 30 min sampling rate was defined for reading all sensors, totaling 48 records per day. After reading the sensors, the ESP32 microcontroller connects to the server and transmits the data, which are stored and made available for viewing via a web page and a mobile application developed specifically for real-time graphical tracking of the monitored parameters.

### 2.10 Real-Time Data Acquisition from the Thermal Mass Flow Meter

The thermal mass flow meter provides a connection option via the Modbus protocol with RS-485 communication. Typically, register addresses are pre-established by the manufacturer and detailed in its datasheet, allowing access to the operating variables available in the meter.

The protocol can be implemented over different types of physical media such as RS-232, RS-485, and Ethernet. In the case of Modbus RTU (Remote Terminal Unit), communication is carried out via RS-485, a serial communication standard that enables data transmission over long distances and in noisy industrial environments.

For this work, the focus was on using a Modbus RTU to communicate with the mass flow meter. The meter’s configuration allows connection via Modbus, where process variables such as instantaneous flow rate, totalized flow, and pressure values can be stored in registers. When requested by a master device—such as a microcontroller, PLC (Programmable Logic Controller), or IoT (Internet of Things) device—these values can be transmitted and stored in databases. This enables real-time analysis through SCADA (Supervisory Control and Data Acquisition) or HMI (Human–Machine Interface) systems. To establish connectivity between the flow meter and the ESP32 microcontroller, a TTL-to-RS-485 data converter was used.

### 2.11 Real-Time Data Acquisition from the Gascard Characterization Board

For the connection between the ESP32 microcontroller and the biogas characterization board (Gascard NG sensor), RS-232 communication was implemented. To this end, a MAX3232 mini RS-232-to-TTL converter module was used.

The Gascard NG infrared gas sensor transmits acquired data continuously while powered, at a baud rate of 57,600 bits per second, and the MAX3232 converter proved effective as an interface with the Gascard NG infrared gas sensor in laboratory tests, delivering consistent high-frequency readings.

### 2.12 Power Board Design and Assembly

As the power supply for the entire system, a 24 V DC source was implemented to provide the necessary power to the sensors and microcontrollers. The supply was installed in an enclosure, and together with it, a board was designed containing two step-down voltage regulator modules responsible for reducing 24 to 5 V. This enables powering the sensors and microcontrollers used in this work, as well as the biogas flow meter and the methane characterization board, ensuring that all components operate within their technical specifications.

### 2.13 Web Server

Due to the need for greater flexibility and control, a custom web server was developed in Node.js and MongoDB, replacing third-party services such as Firebase. The architecture enables centralized processing and storage, simultaneously serving multiple sources (mobile app, web platform, and data acquisition board) and applying business rules to ensure operational integrity.

Security was implemented with JSON Web Tokens (JWT). For communication with the monitoring board (ESP32, dual-core Tensilica LX6, 240 MHz), ES256 (ECDSA with P-256 and SHA-256) is used, which is more efficient than RSA (Rivest–Shamir–Adleman) on resource-constrained devices. For server ↔ web platforms and server ↔ apps, RS256 (RSA with SHA-256) is employed, which is widely adopted in the industry and therefore offers greater compatibility. All traffic is carried over HTTPS (HTTP over TLS/SSL), and tokens use Base64URL encoding per the JWT standard, ensuring integrity and authenticity via digital signatures and confidentiality in transit.

The server operates in a dual role, serving static content (HTML, CSS, JavaScript) and handling API requests, with centralized access via the domain uasb.com.br, protected by SSL/TLS, which ensures security, consistency, and unified management of interactions with different clients.

## 3 Results and Discussion

To support the operational validation of the IoT system under continuous operation, data transmission reliability to the cloud server, communication latency, and self-recovery behavior under connectivity and power disturbances were evaluated. The 30-day operational tests confirmed the integrity of data transmitted via Wi-Fi, with no losses attributable to communication failures. In a seven-day continuous trial, comprising 336 packet transmissions at 30 min intervals, a 100% success rate was achieved. The observed average latency was approximately 1.77 s, which is suitable for real-time monitoring applications. Although higher than benchmark values reported for critical IoT applications, such as emergency response (<450 ms)^16^ or latency-sensitive industrial networks (<300 ms),^17^ the performance remains within the acceptable range for environmental monitoring (1–10 s), as discussed by Dangana et al.^18^ Recent studies also indicate that edge computing architectures can measurably reduce latency, with reductions of up to 60–70% in certain scenarios.^19^

The system demonstrated high fault robustness: following intentional network disconnections and router shutdowns, reconnection occurred autonomously; power interruptions did not compromise the operation, as the microcontroller restarted correctly and resumed data transmission. This behavior is consistent with self-recovery mechanisms described in resilient IoT architectures by Almohri et al.^20^ In contrast, low-cost academic solutions, such as those described by Trevathan and Schmidtke,^7^ although affordable, exhibit lower fault tolerance and still lack large-scale field validation. The proposed modular architecture—low-cost (<USD 5000) and based on open hardware—therefore represents a technical advancement for decentralized applications, especially in regions with limited connectivity.

### 3.1 Validation

Operational validation in the bench-scale UASB reactor indicated that the proposed architecture supports continuous monitoring, with reliable acquisition and transmission of key variables (biogas flow rate; CH_4_/CO_2_; internal temperature), in addition to tolerance to connectivity and power disturbances. The instrumentation operated stably both in Wi-Fi communication and in the dedicated serial links, maintaining continuity of readings and temporal and internal coherence among channels, which is a necessary condition for real-time monitoring applications.

Beyond the robustness of data acquisition and communication, validation was corroborated by the agreement between the measured time series and independent estimates based on the removed organic load (COD removed, normalized to STP), as evidenced by the error indicators (MAE and MAPE) and by the distribution of valid measurements presented in the following section. In this context, the system demonstrates operational fitness for purpose, namely, continuous monitoring and decision support, with potential for decentralized application at costs lower than those of commercial solutions.

### 3.2 Biogas Flow/Composition and NDIR Sensor Performance

Measurements with the biogas thermal mass flow meter indicated 42.84–76.16 NL·d^–1^ (flow), while the characterization module recorded 53.31–88.0% (%CH_4_). The estimates corresponded to 45.18–74.72 NL·d^–1^ (flow). For methane content, the comparison focused on the measured %CH_4_ time series against the assumed compositional range (*K*) used in the stoichiometric conversion, and agreement was summarized by MAE (p.p.) and MAPE. The unpaired dispersion of measurements within the observed ranges is summarized in Figure 4: for flow, *n* = 939, median ≈ 59 NL·d^–1^, and IQR = 8.41 NL·d^–1^; for %CH_4_, *n* = 676, median ≈ 70% v/v, and IQR = 13.93 percentage points. These values support operational variability consistent with UASB systems treating high-fat effluents with measured methane contents within the range reported in the literature. In parallel, temporal agreement was observed between estimated and measured series, with MAE = 6.58 NL·d^–1^ (flow) and 4.69 p.p. (composition), and MAPE = 9.88% (flow) and 6.69% (composition). Taken together, the findings constitute an operational validation of the arrangement (field responsiveness), while the NDIR instrumentation exhibited sensitivity/stability suitable for continuous use, in line with previous studies^12,23,24^ and with plausible %CH_4_ ranges.^4^

**Figure 4:**
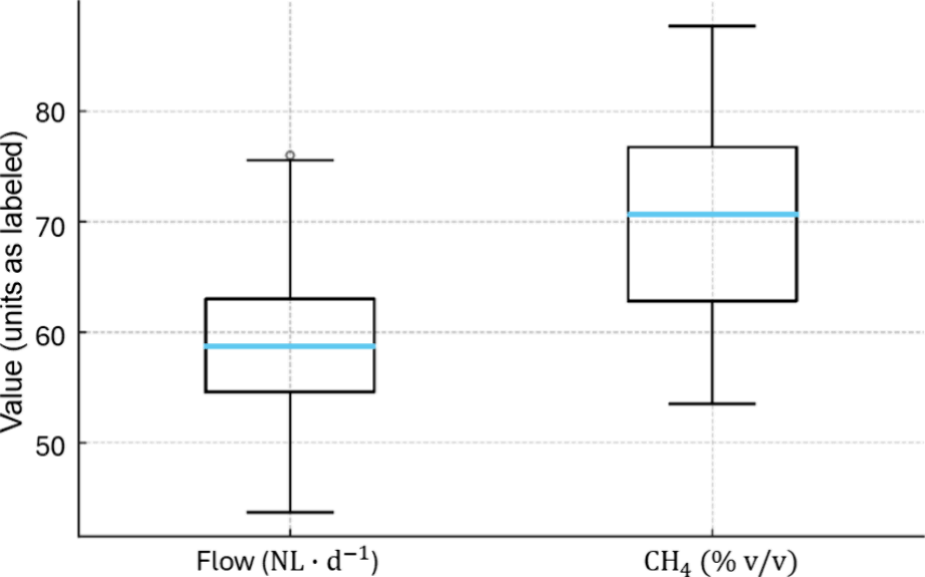
Dispersion of all valid (unpaired) measurements within the observed ranges: flow 42.84–76.16 NL·d^–1^ and CH_4_ 53.31–88.0% (dry gas, STP). Boxplots show the median (line), IQR (Q1–Q3, box), and whiskers = 1.5 × IQR; points beyond these limits are displayed as outliers.

### 3.3 Operational Performance of the Temperature Sensor

The in situ internal temperature of the UASB remained within a narrow mesophilic range throughout the campaign: 22.25 to 27.80 °C (*n* = 321; mean = 25.15 °C; SD = 1.67 °C; CV = 6.65%), consistent with the operational stability typically observed in this range.^2^ From a metrological standpoint, the DS18B20 sensor exhibited stable performance, with virtually zero drift (≈ +0.00003 °C·day^–1^) and low short-term variability consistent with the sensor’s 12-bit quantization (0.0625 °C) and the applied sampling procedure. These findings align with the device specifications (nominal accuracy ±0.5 °C between −10 and +85 °C; configurable resolution up to 0.0625 °C, 12-bit) and support the use of in situ readings as the primary reference for screening and statistical analyses of anaerobic metabolism.^9^ Ex situ readings were used only as traceable spot checks; when cross-comparisons were required, strict temporal pairing was adopted (±5–10 min window) with quantification of bias (95% CI) and limits of agreement.^15^ Under these conditions, the series maintained temporal and internal coherence (across measurement points) and yielded physically and biologically plausible relationships with flow and %CH_4_—i.e., they did not indicate spurious temperature effects where the literature predicts a reduced impact.^2,25^

### 3.4 Comparative Analysis with the Literature and Commercial Systems

Table 2 summarizes the comparison among commercial systems, reported academic solutions, and the system developed in this study. The results indicate that while commercial systems exhibit high robustness, their high cost and proprietary nature limit deployment in decentralized contexts. Academic solutions, although lower-cost, lack practical field validation and show robustness constraints. The proposed system, in turn, combines low cost, fault tolerance, and a modular architecture based on open hardware, favoring its replicability in decentralized facilities, including in regions with limited connectivity.

**Table 2:** Comparison of Commercial Systems, Academic Solutions, and the Proposed System^a^

system	cost	robustness	technical capabilities	replicability
commercial	high (USD 10,000–50,000)	high, dependent on stable infrastructure	flow rate, CH_4_, CO_2_; wired/GSM connectivity	low (proprietary solutions)
academic	low to medium	limited; little field validation	CH_4_, CO_2_, T°; spotty GSM/Wi-Fi	medium (laboratory prototypes)
proposed system	low(<USD 5000)	high; autonomous reconnection and postfailure restart	flow rate, CH_4_, CO_2_, T°, pressure; Wi-Fi + dedicated server	high (modular, open-source)

a Data for the proposed system are from this study. Information on commercial systems was based on manufacturers’ technical literature,^21–23^ and the reported academic solutions were adapted from Acharya et al.^11^

## 4 Final Considerations

The proposed low-cost IoT system—integrating NDIR sensors, ESP32 nodes, and web/mobile interfaces—proved feasible for real-time monitoring of UASB reactors, with a mean latency of 1.77 s, lossless transmissions in a 7-day test (336 sends), and stable operation over 30 days, while costing substantially less than benchmark commercial solutions. Its modular, fault-tolerant architecture (autonomous reconnection and restart) enabled continuous acquisition of biogas flow, CH_4_/CO_2_, and temperature; the latter remained within a narrow mesophilic range (22.25–27.80 °C) with virtually zero sensor drift, supporting the reliability of operational interpretations.

Estimates based on COD removed and normalized to STP showed good temporal agreement with the measured series (MAE_CH4_ ≈ 4.69 percentage points; MAPE_CH4_ ≈ 6.69%; MAE_flow_ ≈ 6.58 NL·d^–1^; MAPE_flow_ ≈ 9.88%), reinforcing the applicability of the setup in decentralized contexts. Taken together, the results point to accessible instrumentation that strengthens process control and biogas energy recovery in sanitation.
